# Supplementary material for: Elucidating the Role of SlBBX31 in Plant Growth and Heat-Stress Resistance in Tomato
Source: Int J Mol Sci. 2024 Aug 27;25(17):9289. doi: 10.3390/ijms25179289 (PMC11395151; doi:10.3390/ijms25179289)
Supplement: Supplementary file 1 [file ijms-25-09289-s001.zip › Supplementary materials.pdf]

Supplementary Materials

Table S1. Primers used in this study.

Name	Primer（5'-3'）
OE-SlBBX31-F	TTCATTTGGAGAGGACACGCTCGAGATGGAACTTCTGTCCTCTAAAC
OE-SlBBX31-R	TATCTCATTAAAGCAGGACTCTAGATTAGGGCGAGGATTCAGC
SlBBX31-SgRNA-F	GATTGAAAGCTTCAAATGAACCGCG
SlBBX31-SgRNA-R	AAACCGCGGTTCATTTGAAGCTTTC
SlBBX31-GFP-F	ACACGGGGGACGAGCTCGGTACCATGGAACTTCTGTCCTCTAAAC
SlBBX31-GFP-R	CTCACCATGGTGTCGACTCTAGAGGGCGAGGATTCAGC
SlBBX31-qPCR-F	TGATACACGGGATGAACAAGAA
SlBBX31-qPCR-R	TGTACAAGGTCGTCAATGGTTA
Actin7-qPCR-F	GTTTGTGACAATGGAACTGGAA
Actin7-qPCR-R	CCAATTGCTGACTATACCATGC
LEA-qPCR-F	CTGGGACAATTGGTGAAAAGTT
LEA-qPCR-R	TCTCTTTCAGCCCTTTCTTCTT
Hsp70-1-qPCR-F	GCTCGAGGGCATCTGTAATC
Hsp70-1-qPCR-R	GAAACCAACTAGTATCTTTATCAACC

Table S2. Expression analysis of *SlBBX* gene family in heat stress transcriptome.

Gene Name	Heat-2h_vs_Heat-0h	Heat-4h_vs_Heat-0h	Heat-12h_vs_Heat-0h	Heat-24h_vs_Heat-0h
SlBBX1	-2.287639131	-2.652365577	-1.588390555	-3.479838589
SlBBX2	-1.221686512	-1.496516884	-1.195637761	-2.84146372
SlBBX3	-0.724906723	-0.643507241	-1.344094741	-0.925013949
SlBBX4	-1.009917009	-1.316756497	-1.756680512	-0.225660156
SlBBX5	1.355508169	0.321749415	1.162339322	-1.523814432
SlBBX6	0.187051725	-0.039308429	-1.198253911	-2.059027571
SlBBX7	0.526141592	0.202336867	1.511407946	2.497816597
SlBBX8	NA	NA	NA	NA
SlBBX9	0.087155865	-0.113017835	0.574355813	0.088488586
SlBBX10	-0.888489212	-2.211293545	-1.826554056	-2.32455
SlBBX11	1.367066405	1.372922058	2.018997158	-0.137383749
SlBBX12	NA	NA	NA	NA
SlBBX13	-1.701953976	-4.323791448	-3.248223254	-5.775489771
SlBBX14	0.291318259	0.224356209	2.42982598	0.428059884
SlBBX15	-0.161447084	-0.526684262	-0.314323951	-1.279625367
SlBBX16	NA	NA	NA	NA
SlBBX17	0.094354266	-0.96022198	-0.241932621	0.980128033
SlBBX18	0.016809962	-0.364961858	-0.013448708	-2.148882408
SlBBX19	-1.304920993	-2.107389095	-1.09240901	0.961823758
SlBBX20	0.271967146	-0.959554379	-0.448573516	-1.033807432
SlBBX21	NA	NA	NA	NA
SlBBX22	-0.020997038	-0.346747011	0.444179427	-0.352450167
SlBBX23	-0.361877338	-0.371962593	-0.338072696	-3.411108729
SlBBX24	0.426389733	-0.434499778	0.436823843	0.052635616
SlBBX25	NA	NA	NA	NA
SlBBX26	0.676932883	-0.394673509	0.151198049	-0.093638054
SlBBX27	-1.350460517	-1.644867448	-1.070779024	-1.276439977
SlBBX28	NA	NA	NA	NA
SlBBX29	-1.627730361	-1.618231745	-1.46903579	-3.339300946
SlBBX30	-1.077991186	-1.079242686	-1.149262048	-0.565630211
SlBBX31	0.71005904	1.224943232	1.060280437	2.718083218

[a] The log_2FC value was calculated based on the FPKM value of each gene at 0 h.

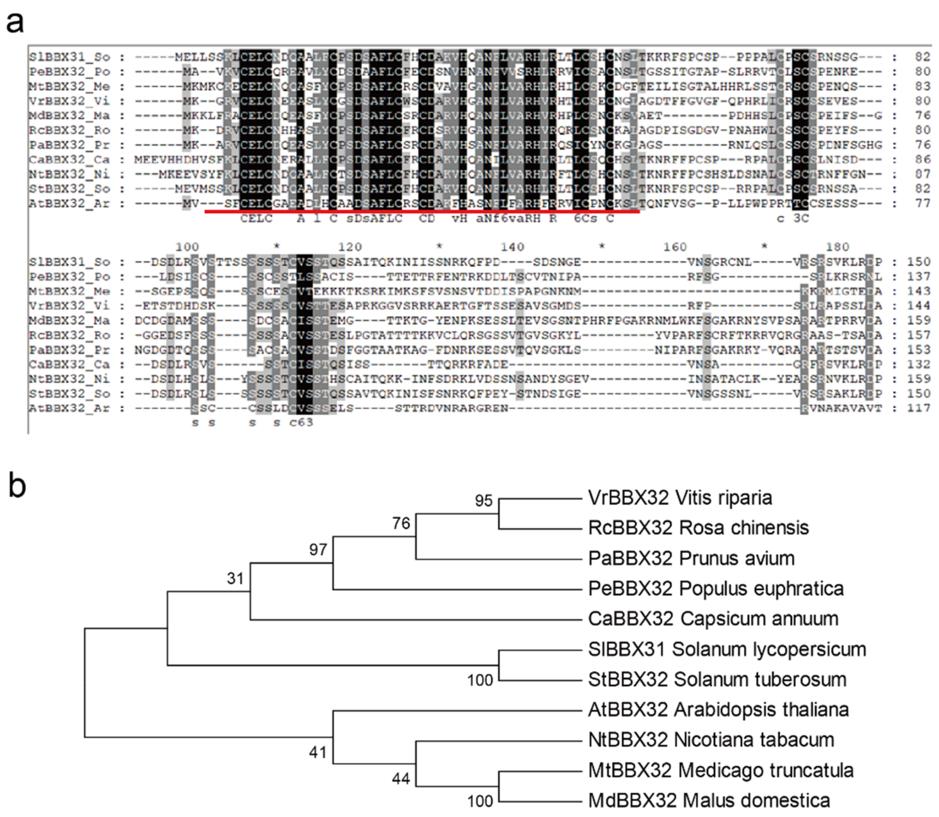

Figure S1. Phylogenetic analysis of SlBBX31. (a) Alignment of amino acid sequence of SlBBX31 with homologous sequences in different species. The conserved B-Box domain is in the red area. (b) Phylogenetic tree analysis of SlBBX31 protein and other homologous proteins.

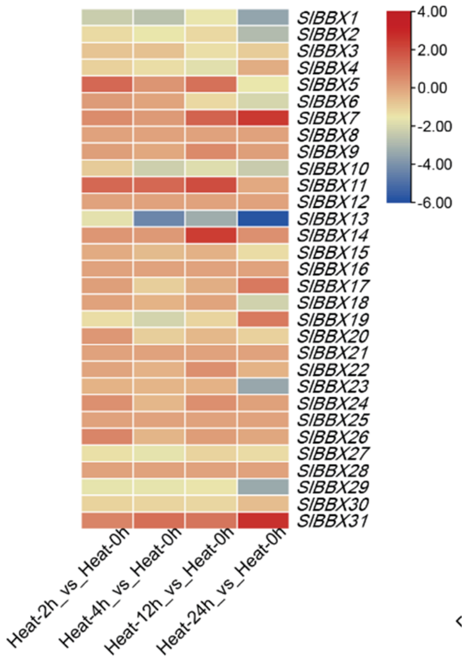

Figure S2. Expression analysis of *SlBBX* gene family in heat stresses.

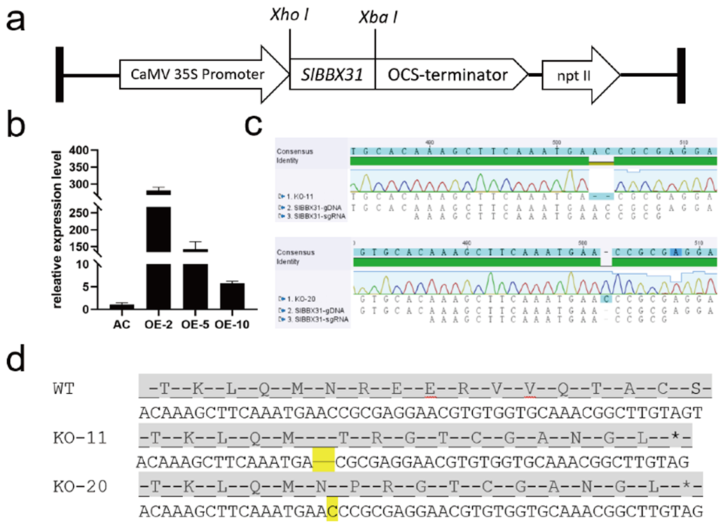

Figure S3. Identification of overexpression and knockout lines of *SlBBX31*. (a) Schematic diagram of the overexpression vector of *SlBBX31*. (b) The relative expression level of *SlBBX31* in overexpression lines. (c) Detection of two homozygous knockout transgenic plants. The KO-11 lines had a double base deletion, while the KO-20 lines had a single base insertion. (d) Mutated amino acid and nucleotide sequences of KO-11 and KO-20 lines. Due to a mutation, translation is prematurely terminated, leading to the generation of truncated proteins. The highlighted position indicates the site of this mutation.
